# Hepatitis E Virus Induces Hepatocyte Apoptosis via Mitochondrial Pathway in Mongolian Gerbils

**DOI:** 10.3389/fmicb.2018.00460

**Published:** 2018-03-16

**Authors:** Yifei Yang, Ruihan Shi, Majid H. Soomro, Fengjiao Hu, Fang Du, Ruiping She

**Affiliations:** ^1^Institute of Chinese Materia Medica, China Academy of Chinese Medical Sciences, Beijing, China; ^2^Laboratory of Animal Pathology and Public Health, Key Laboratory of Zoonosis of Ministry of Agriculture, College of Veterinary Medicine, China Agricultural University, Beijing, China

**Keywords:** mitochondrion, hepatitis E virus, hepatocyte, apoptosis, injury

## Abstract

Previous studies demonstrated that Mongolian gerbils can be infected by hepatitis E virus (HEV), which induces the hepatic injury. Here, the mitochondria in hepatocytes from HEV-infected gerbils were considerably swollen, thin cristae. After HEV infection, the activity of superoxide dismutase significantly decreased (*p* < 0.01), while malondialdehyde concentrations significantly increased, compared with those in the control group (*p* < 0.01). Adenosine triphosphatase levels decreased significantly in the hepatocyte of the inoculated groups, compared with those in control group (*p* < 0.05) at days 21, 28, 42 post-inoculation (dpi) as well. Furthermore, the levels of ATP synthetase ATP5A1 significantly decreased during HEV infection, compared with those in the control group (*p* < 0.05). According to the TdT mediated dUTP nick end labeling (TUNEL) detection, TUNEL positive hepatocytes increased in the inoculated group, compared with that in the control group (*p* < 0.05). Up-regulation of the mitochondrion-mediated apoptosis regulating proteins, Bax and Bcl-2, in the HEV-infected gerbils (*p* < 0.05) was observed. However, cytochrome c levels in mitochondria decreased, while this molecule was detected in the cytoplasm of the infected animals, in contrast to that in the control group. Apaf-1, and active caspase-9 and -3 levels were shown to be significantly higher in the inoculated group compared with those in the control group (*p* < 0.05). Taken together, our results demonstrated that HEV infection induces hepatocyte injuries and activity of the mitochondrial apoptotic pathway, which trigger the hepatocyte apoptosis in Mongolian gerbils.

## Introduction

Hepatitis E virus induces the development of hepatitis in many organisms, including humans. HEV is a non-enveloped, positive-sense, single-stranded 7.2-kb RNA virus belonging to the family Hepeviridae (Smith et al., 2014). It contains three ORFs (Panda et al., 2007), and HEVs isolated from humans, pigs, deer, wild boars, rabbits, camels, and mongooses belong to the Orthohepevirus A group, while HEV isolated from chickens belongs to Orthohepevirus B, from rats to Orthohepevirus C, and from bats to Orthohepevirus D group (Smith et al., 2014). HEV infections represent an important public health problem and may lead to mortality and morbidity, especially in the developing countries.

Mitochondria are now recognized as the site of respiration-dependent ATP synthesis. Furthermore, mitochondria fulfill many other important functions in eukaryotic cells and play a role in calcium storage, amino-acid metabolism, iron–sulfur cluster synthesis, lipid metabolism, and programmed cell death (Lill and Mühlenhoff, 2008; Martinou and Youle, 2011; Kulawiak et al., 2013).

Mitochondrial apoptosis pathway can be induced by mitochondrial injury and it is affected by the Bcl-2 family members (Degli Esposti et al., 2012; Wang, 2014). Ultimately, the effector caspases are activated, cleaving and degrading cell structures, which results in the release of the apoptotic products into the circulation (Wang, 2014). Viruses, drugs, and their reactive metabolites affect mitochondrial respiratory chain, causing a series of harmful responses, such as ATP depletion, mitochondrial DNA damage, and an increase in the permeability of the mitochondrial membranes (Huang et al., 2010; Lucena et al., 2010). Mitochondrial membrane permeabilization, release of cytochrome c, which then binds to Apaf-1, and this complex trigger its oligomerization, forming an apoptosome and recruiting pro-caspase-9. The activated pro-caspase-9 subsequently activates caspase-3 that cleaved target proteins, leading to the cell apoptosis (Degli Esposti et al., 2012; Wang, 2014).

Superoxide dismutase is involved in the cellular defense against the harmful oxygen species (Pandey et al., 2003), while an increase in MDA production induces the oxidative stress (Monteiro et al., 2006; Kavitha and Rao, 2008; Tellez-Banuelos et al., 2009). Therefore, a decrease in the SOD activity and increase in the MDA levels represent the markers of hepatocyte oxidative stress, indicating that the critical oxidant -antioxidant balance is disrupted, which can result in hepatocellular ultrastructure alterations and induced apoptosis (Xu et al., 2009). Furthermore, a previous study demonstrated that the oxidative stress may lead to cellular apoptosis (Sinha et al., 2013). The relationship between SOD or MDA and the HEV infection has been rarely investigated. However, in a previous study, significant increase in MDA levels in hepatitis C virus (HCV) infected patients was observed, and it was demonstrated to be significantly correlated with the HCV RNA viral load. Additionally, MDA levels were proposed to be used as the markers for the monitoring of oxidative stress during HCV infections (Tawadrous et al., 2012).

Previously, it was demonstrated that the liver-to-body ration increases following the inoculation of animals with HEV (Li et al., 2009; Yang et al., 2015). A significant increase in AST, ALT, and TBIL levels in the sera of Mongolian gerbils was observed, while HEV IgG was detected at day 21 post-inoculation (dpi) (Li et al., 2009; Yang et al., 2015). Ultrastructure analyses demonstrated that the mitochondrial morphology was altered during HEV infection. However, the molecular mechanisms underlying this injury have not been elucidated. In order to understand the molecular mechanisms of HEV-associated hepatocyte mitochondrial injury pathogenesis, we analyzed the ultrastructure and functional alterations of mitochondria, using Mongolian gerbils infected with HEV.

## Materials and Methods

### Ethics Statement

This study was carried out in accordance with the recommendations of ethical guidelines and regulations for the use of laboratory animals by the Animal Care and Use Committee of China Agricultural University (CAU) (approval number: 20140115-089). The protocol was approved by the CAU Animal Care and Use Committee.

### HEV Strain

The strain of HEV, a genotype 4 virus, was derived from the liver sample from a SPF swine infected with HEV (CHN-HB-HD-L2, GenBank accession number KM024042). A 10% (g/mL) homogenate of HEV-positive liver with a titer of 6.57 × 10^8^ genome equivalents (GE) per mL was prepared (Huang et al., 2009) and tittered using real-time PCR, as previously described (Jothikumar et al., 2006; Zhao et al., 2007; Zwettler et al., 2012; Yang et al., 2015). The obtained homogenate was stored at -86°C. All experiments involving viruses were performed in the Animal Bio-safety Level-2 (ABSL-2) facilities. All materials involving viruses were sterilized by autoclaving after use to ensure the inactivation of the viruses.

### Animals

Eighty-four SPF male Mongolian gerbils (Meriones unguiculatus, body weight: 50–60 g; age: 8–10 weeks), were purchased from the Department of Experimental Animal Sciences of Capital Medical University (Beijing, China). To avoid extensive stress to the animals, all experiments were performed following an acclimation period of 3 days after their-arrival to our facility. All animals used in this study were confirmed to be negative for HEV antibodies by ELISA and there were no HEV antigens detected in their sera or fecal samples, according to the nested-PCR analyses, as previously described (Yang et al., 2015). All animals were housed in the SPF facilities.

### Experimental Design

The gerbils were randomized into two groups. Each gerbil in the infected group was intraperitoneally injected with 0.1 mL of the viral homogenate. Gerbils in the control group were inoculated with the equal volume of homogenate from an SPF swine liver that tested negative for HEV, as previously described (Yang et al., 2015). All gerbils were given food and water *ad libitum* during the experimental period.

### Sampling

Six gerbils were euthanized to perform necropsy at 0, 7, 14, 21, 28, 42, and 56 dpi. Fresh frozen liver tissues were collected to analyze SOD, MDA, and ATPase levels, western-blot and stored at -86°C. Additional liver tissue was collected and fixed in neutral 4% paraformaldehyde for 7 days. The preparation of sections for the TUNEL and immunohistochemical (IHC) analyses were performed, as previous described (Yang et al., 2015). Some of the other tissues were fixed in 2.5% (v/v) glutaraldehyde-polyoxymethylene solution for 6–8 h and analyzed using the TEM (Yang et al., 2015).

### TEM Analyses

For the TEM analyses, liver samples were performed as previously described (Yang et al., 2015). These ultra-thin sections were observed using a JEM 100CX TEM.

### SOD, MDA, and ATPase Levels in Hepatocytes

The obtained liver homogenates were centrifuged at 3000 rpm for 10 min and the supernatants were used to detect SOD and MDA levels. The activity of SOD in hepatocyte was detected by using total SOD (T-SOD) assay kit (hydroxylamine method) (Nanjing Jiancheng Bioengineering Institute). MDA concentrations were detected by using the MDA assay kit (TBA method) (Nanjing Jiancheng Bioengineering Institute), while the ATPase activity was detected by ultramicro-determination ATPase assay kit (Beijing Sino-UK Institute of Biological Technology).

### TUNEL Staining

The apoptotic cells in the livers were detected using TUNEL staining. An *in situ* detection kit was used according to the manufacturer’s instructions (In Situ Cell Death Detection Kit, POD, 11684817910; Roche Biochemicals). The apoptotic indices were calculated as the ratio of positively stained hepatocytes in 100 hepatocytes observed with in one field. Five fields per one sample were analyzed at 400× magnification).

### IHC Assays

Immunohistochemical staining was performed using a commercial kit, according to the manufacturer’s instructions (ZSGB-BIO, Beijing, China). Primary antibodies used in this study were anti-Apaf-1 (1:200, BA2373), anti-Bax (1:300, BA0315), anti-Bcl-2 (1:200, BA0412) and anti-active caspase-3 (1:300, BA3968), anti-active caspase-9 (1:300, BA0690), all obtained from Boster, Co., Ltd., Beijing, China.

Apaf-1, Bax, Bcl-2, and active caspase-3, active caspase-9 positive signals were observed as brown or yellow granular masses in cells, and their intensities were measured using Motic Med 6.0 CMIAS Image Analysis System (Motic China Group, Co., Ltd., China). A total of 15 fields per gerbil (three fields per section, five sections per gerbil, 400× magnification) were randomly selected and analyzed. The positive staining intensity was calculated as the ratio of the stained area to the total field assessed (Du et al., 2015).

### Western-Blot Analysis

Small amount of Mongolian gerbil livers obtained from each group were homogenized in the lysis buffer (7 M urea, 2 M thiourea, 4% CHAPS detergent, 1% DTT, 400 mM Tris-base, and 1 mM PMSF) (Yang et al., 2015). After centrifugation at 12000 rpm for 20 min at 4°C, the supernatants were collected and used as the tissue lysate. Hepatocyte cytoplasmic and mitochondrial proteins were extracted from the liver using Cytoplasmic and Mitochondria Protein Extraction Kit (Sangon Bio-Tech, China) to determine the levels of cytochrome c in both the cytoplasm and mitochondria of hepatocytes (Pan et al., 2014). Protein concentrations were determined by Nanodrop2000 spectrophotometer (Thermo, United States). The proteins were separated using sodium dodecyl sulfate polyacrylamide gel electrophoresis (SDS-PAGE) under reducing conditions, and electro-blotted onto polyvinylidene fluoride (PVDF) membranes, as previous described (Yang et al., 2015). The primary antibodies were used including cytochrome c, Apaf-1, Bax, Bcl-2, active caspase-3, active caspase-9, ATP5A1, GAPDH, and β-actin.

### Statistical Analysis

Experimental data were analyzed using a one-way analysis of variance (ANOVA) with the SPSS20.0 statistical program. The equality of variance was tested and *post hoc* tests were performed as well. *p* < 0.05 was considered statistically significant.

## Results

### HEV Infection Induces Mitochondrial Lesion Development

**FIGURE 1:**
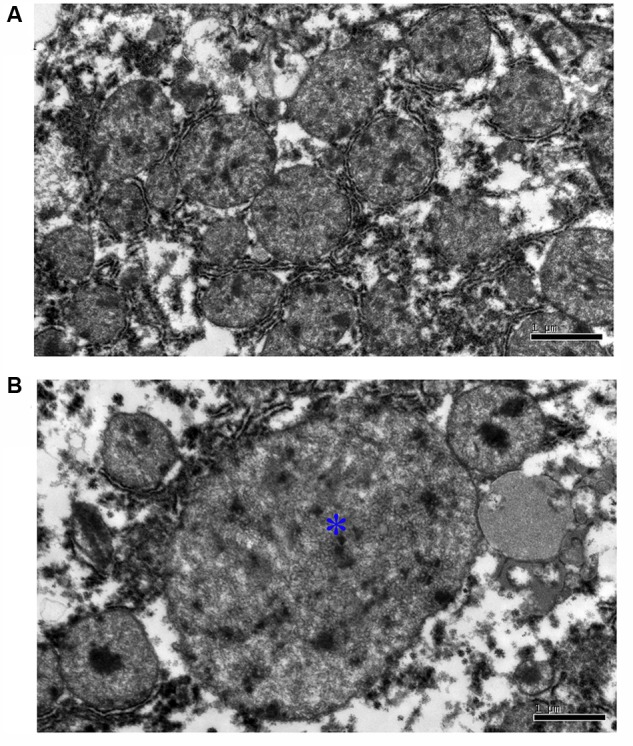
Ultrastructural pathological analysis of mitochondrion in Mongolian gerbil livers. **(A)** No apparent pathological change was observed in the mitochondrion in control group. **(B)** The mitochondria were considerably swollen and were shown to have thin cristae (^∗^).

In the control group, no apparent pathological mitochondrial changes were observed (**Figure 1A**). However, in the inoculated group, the mitochondria were considerably swollen and were shown to have thin cristae (**Figure 1B**).

### HEV Infection Leads to SOD Activity Decrease and MDA Concentration Increase

**FIGURE 2:**
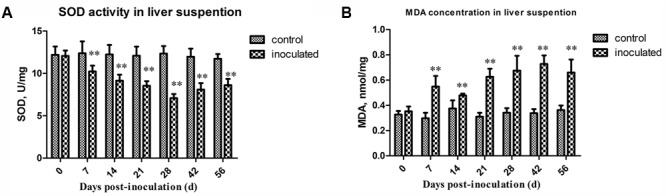
Changes in activity of SOD and concentration of MDA. **(A)** The activity of SOD decreased significantly in inoculated group compared to that of the control group (*p* < 0.01) at 7, 14, 21, 28, 42, and 56 dpi. And at 28 dpi, the activity of SOD was at the lowest level, 7.08 ± 0.89 U/mg. **(B)** MDA concentration significantly increased in livers of inoculated group after HEV infection than the control group (*p* < 0.01). And the concentration of MDA was at the peak at 42 dpi, 0.73 ± 0.07 nmol/mg. ^∗^*p* < 0.05, ^∗∗^*p* < 0.01.

Superoxide dismutase activity and MDA concentration in the investigated animals are presented in **Table 1**. The activity of SOD was shown to decreased significantly in the inoculated group at 7, 14, 21, 28, 42, and 56 dpi, compared with that in the control group (*p* < 0.01) (**Figure 2A**). At 28 dpi, SOD activity was shown to be at the lowest level, 7.08 ± 0.89 U/mg, in the HEV-infected animals. In contrast, MDA concentrations significantly increased in the livers of the HEV-infected gerbils in comparison with those detected in the control group (*p* < 0.01; **Figure 2B**). MDA concentration peaked at 42 dpi (0.73 ± 0.07 nmol/mg).

**Table 1:** Activity of SOD and concentration of MDA in experimentally infected Mongolian gerbils.

DPI	0 dpi		7 dpi		14 dpi		21 dpi		28 dpi		42 dpi		56 dpi	
Group	Control	Inoculated	Control	Inoculated	Control	Inoculated	Control	Inoculated	Control	Inoculated	Control	Inoculated	Control	Inoculated
SOD (U/mg)	12.1887 ± 0.9970^a^	12.0730 ± 0.6350	12.3808 ± 1.3957	10.2327 ± 0.6910^∗∗^	12.2483 ± 1.1259	9.1463 ± 0.7131^∗∗^	12.0985 ± 1.0701	8.5457 ± 0.5367^∗∗^	12.3445 ± 0.8869	7.0803 ± 0.4894^∗∗^	11.9640 ± 0.9720	8.0887 ± 0.7660^∗∗^	11.7317 ± 0.5635	8.6167 ± 0.7364^∗∗^
MDA (nmol/ mg)	0.3267 ± 0.0287	0.3522 ± 0.0387	0.2983 ± 0.0426	0.5487 ± 0.0844^∗∗^	0.3758 ± 0.0635	0.4788 ± 0.0145^∗∗^	0.3100 ± 0.0309	0.6263 ± 0.0643^∗∗^	0.3413 ± 0.0370	0.6750 ± 0.1176^∗∗^	0.3382 ± 0.0323	0.7277 ± 0.0673^∗∗^	0.3630 ± 0.0361	0.6607 ± 0.1022^∗∗^

*^*a*^Data are means ± SD; ^∗^*p* < 0.05 and ^∗∗^*p* < 0.01.*

### HEV Infection Decreases ATPase Activity and ATP5A1 Expression in Hepatocytes

**FIGURE 3:**
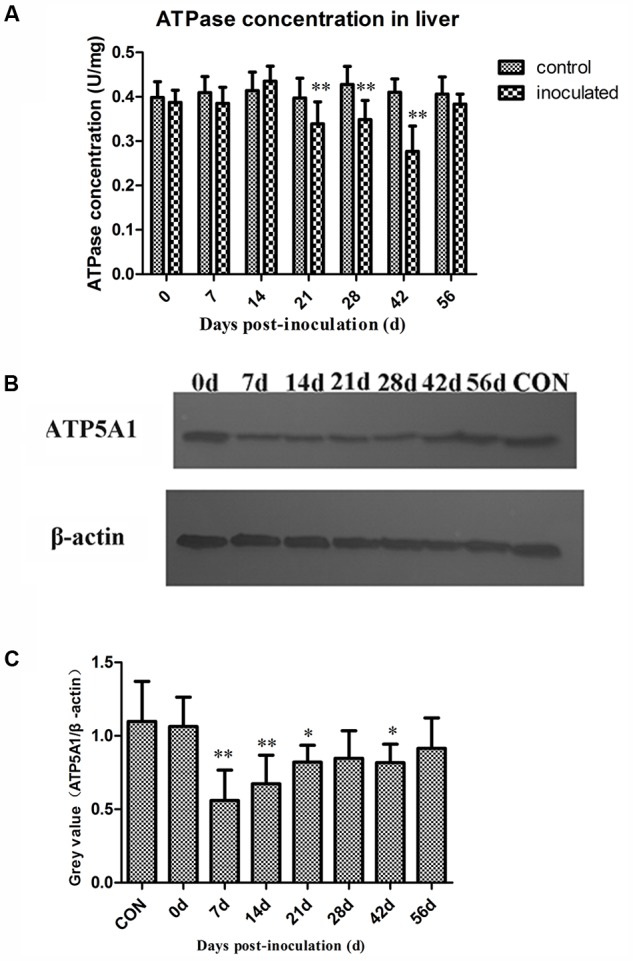
Changes in activity of ATPase and ATP sythetase ATP5A1. **(A)** The activity ATPase in hepatocyte decreased significantly in inoculated group compared with the control group at 21, 28, and 42 dpi (*p* < 0.01),^∗^*p* < 0.05, ^∗∗^*p* < 0.01. **(B)** Western-blot analysis of ATP5A1 in hepatocyte of control group and inoculated group gerbils. **(C)** A semi-quantitative analysis of relative expression of ATP5A1/β-actin.

The obtained ATPase activity levels are presented in **Table 2**. At 0, 7, and 14 dpi, no differences in the ATPase activity between the control and inoculated groups were observed. However, ATPase activity in hepatocytes significantly decreased in the inoculated group at 21, 28, and 42 dpi, compared with that in the control group (^∗^*p* < 0.05 and ^∗∗^*p* < 0.01, respectively; **Figure 3A**). Western-blot analyses demonstrated that the expression level of ATP5A1 decreased at 7, 14, 21, and 42 dpi, compared with those in the control group (^∗^*p* < 0.05 and ^∗∗^*p* < 0.01, respectively; **Figures 3B,C**).

**Table 2:** Concentration of ATPase in experimentally infected Mongolian gerbils.

DPI	0 dpi		7 dpi		14 dpi		21 dpi		28 dpi		42 dpi		56 dpi	
Group	Control	Inoculated	Control	Inoculated	Control	Inoculated	Control	Inoculated	Control	Inoculated	Control	Inoculated	Control	Inoculated
ATPase (U/mg)	0.3987 ± 0.0352^a^	0.3874 ± 0.0274	0.4093 ± 0.0363	0.3852 ± 0.0361	0.4138 ± 0.0417	0.4350 ± 0.0339	0.3973 ± 0.0446	0.3388 ± 0.0498^∗∗^	0.4275 ± 0.0412	0.3487 ± 0.0433^∗∗^	0.4103 ± 0.0299	0.2768 ± 0.0569^∗∗^	0.4062 ± 0.0384	0.3835 ± 0.0227

*^*a*^Data are means ± SD; ^∗^*p* < 0.05 and ^∗∗^*p* < 0.01.*

### HEV Infection Induces Hepatocyte Apoptosis

**FIGURE 4:**
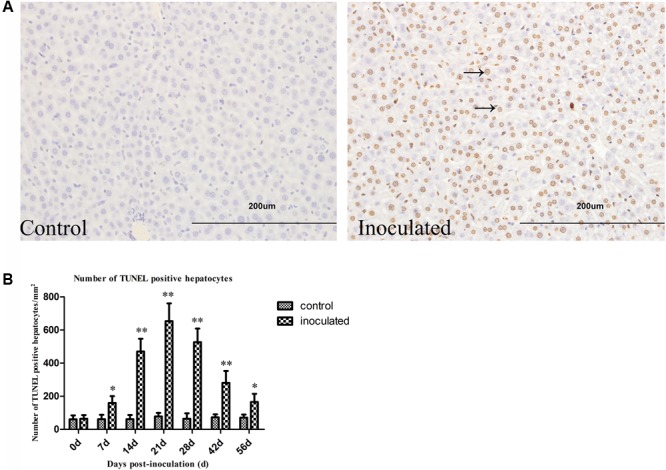
Hepatitis E virus infection induced TUNEL positive hepatocytes increased. **(A)** Representative pictures of TUNEL staining (arrow indicates TUNEL-positive hepatocytes nuclear stained in brown). **(B)** Quantitative analysis of number of hepatocyte apoptosis (/mm^2^). ^∗^*p* < 0.05, ^∗∗^*p* < 0.01.

As presented in **Figures 4A,B**, the number of TUNEL-positive hepatocytes (/mm^2^) was significantly increased in the inoculated group compared with that in the control group (^∗^*p* < 0.05 and ^∗∗^*p* < 0.01, respectively). The number of TUNEL-positive hepatocytes was shown to increase at 7 dpi, and it peaked at 21 dpi.

### HEV Infection Induces the Expression of Proteins Regulating Mitochondrion Mediated Apoptosis

**FIGURE 5:**
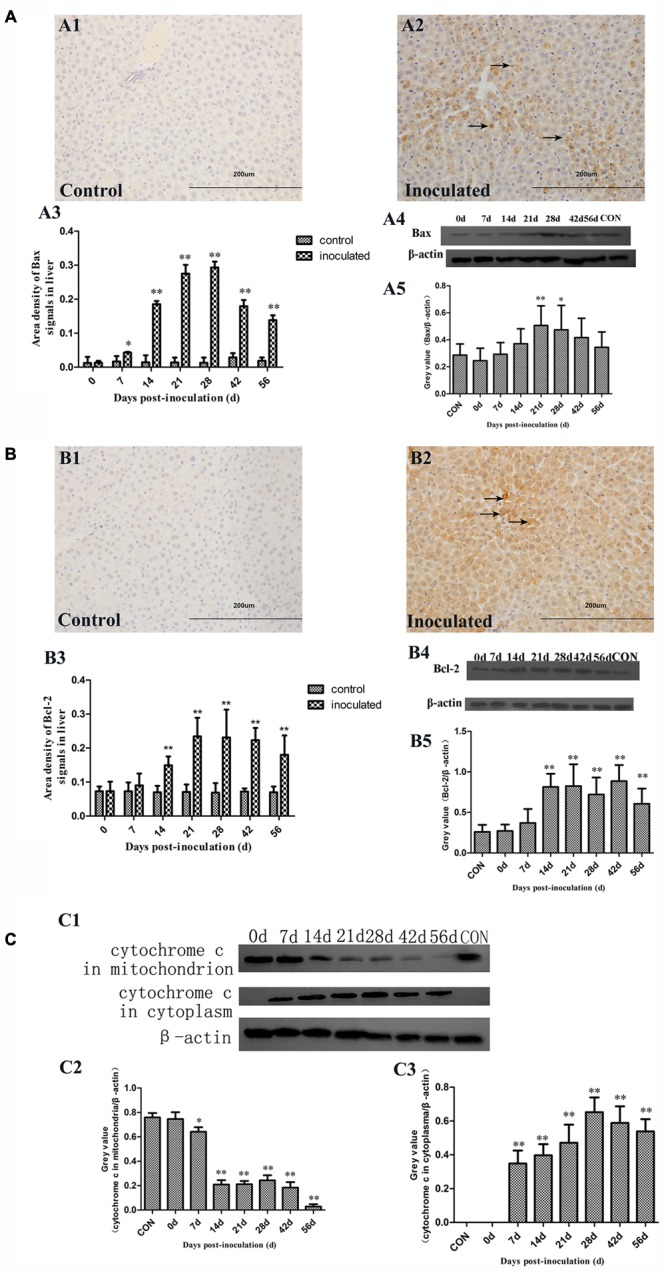
Immunohistochemical and western-blot analysis of Bax, Bcl-2, and cytochrome c proteins. **(A1)** IHC analysis of livers in control group, **(A2)** IHC analysis of livers in inoculated group. The primary antibody was Bax antibody, and positive signals were observed in the cytoplasm of hepatocyte (→); **(A3)** A semi-quantitative analysis of the ratio of Bax positive staining to the total field; **(A4)** Western-blot analysis of Bax in hepatocyte of control group and inoculated group gerbils; **(A5)** A semi-quantitative analysis of relative expression of Bax/β-actin. **(B1)** IHC analysis of livers in control group, **(B2)** IHC analysis of livers in inoculated group. The primary antibody was Bcl-2 antibody, and the positive signals were observed in the cytoplasm of hepatocyte (→); **(B3)** A semi-quantitative analysis of the ratio of Bcl-2 positive staining to the total field; **(B4)** Western-blot analysis of Bcl-2 in hepatocyte of control group and inoculated group gerbils; **(B5)** A semi-quantitative analysis of relative expression of Bcl-2/β-actin. **(C1)** Western-blot analysis of cytochrome c in mitochondrion and cytoplasm of hepatocyte of control group and inoculated group gerbils. **(C2)** A semi-quantitative analysis of relative expression of cytochrome c in mitochondria/β-actin. **(C3)** A semi-quantitative analysis of relative expression of cytochrome c in cytoplasm/β-actin. ^∗^*p* < 0.05, ^∗∗^*p* < 0.01.

Immunohistochemical staining demonstrated that Bax and Bcl-2 levels considerably increased in the cytoplasm of the hepatocytes in inoculated groups (**Figures 5A1,A2,B1,B2**). This protein expression up-regulation was confirmed by using the semi-quantitative analyses (**Figures 5A3,B3**). Furthermore, the ratio of Bcl-2 to Bax was calculated from the results obtained in the semi-quantitative analyses and they are presented in **Table 3**. In the inoculated group, Bcl-2 to Bax ratio was below 1 at 14, 21, and 28 dpi, however, in the control group, these ratios were shown to be above 1. Western-blot analyses demonstrated that Bax and Bcl-2 expression levels in the inoculated group were significantly higher than with those in the control group (^∗^*p* < 0.05 and ^∗∗^*p* < 0.01; **Figures 5A4,A5,B4,B5**).

**Table 3:** Bcl-2 to Bax ratio in experimentally infected Mongolian gerbils.

DPI	0 dpi		7 dpi		14 dpi		21 dpi		28 dpi		42 dpi		56 dpi	
Group	Control	Inoculated	Control	Inoculated	Control	Inoculated	Control	Inoculated	Control	Inoculated	Control	Inoculated	Control	Inoculated
Bcl-2: Bax ratio	6.1	5.6	4.4	2.1	4.9	**0.81**	5.1	**0.85**	5.4	**0.79**	2.5	1.25	3.7	1.31

*Bcl-2 to Bax ratio was below 1 at 14, 21, and 28 dpi in the inoculated group.*

Therefore, we analyzed cytochrome c levels in the cytoplasm and mitochondria (**Figure 5C**). Cytochrome c levels in the mitochondria were shown to decrease between 14 and 56 dpi in the inoculated group. In cytoplasm, cytochrome c was initially detected at 7 dpi until the end of the experiment, but it was undetectable at 0 dpi in inoculated group and control group (**Figure 5C**).

**FIGURE 6:**
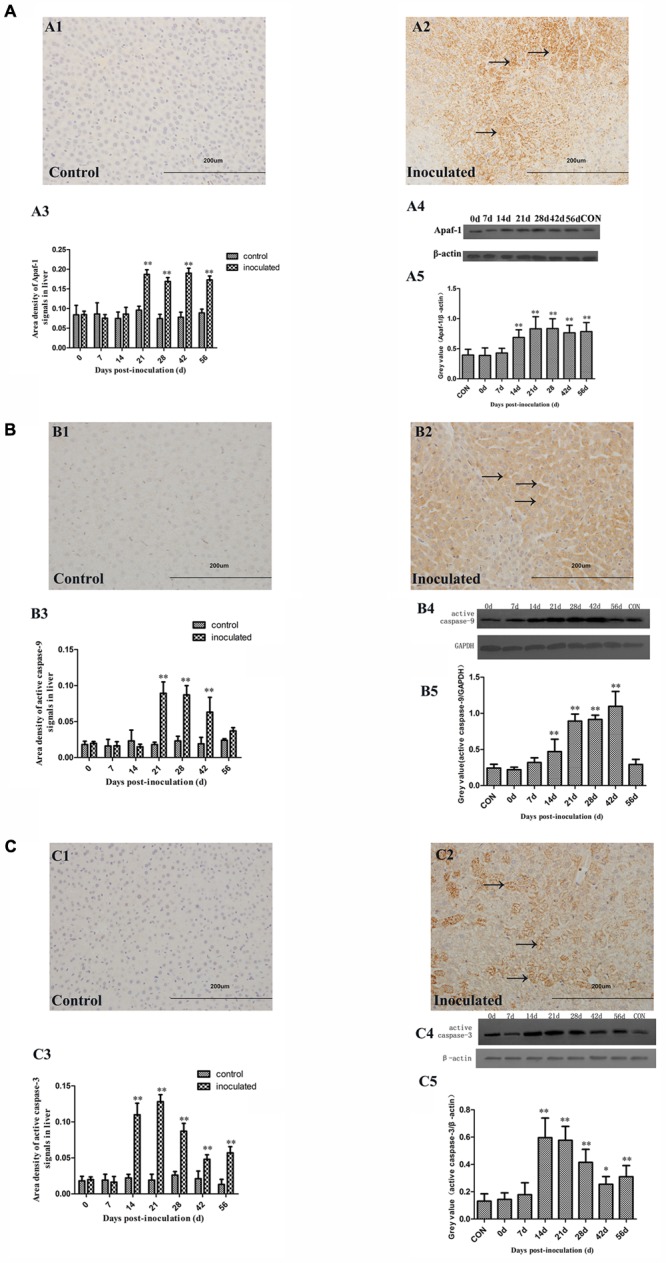
Immunohistochemical and western-blot analysis of Apaf-1, active caspase-9, and active caspase-3 proteins. **(A1)** IHC analysis of livers in control group, **(A2)** IHC analysis of livers in inoculated group. The primary antibody was Apaf-1 antibody, and the positive signals were observed in the cytoplasm of hepatocyte (→); **(A3)** A semi-quantitative analysis of the ratio of Apaf-1 positive staining to the total field; **(A4)** Western-blot analysis of Apaf-1 protein in hepatocyte of control group and inoculated group gerbils; (**A5**) A semi-quantitative analysis of relative expression of Apaf-1/β-actin. **(B1)** IHC analysis of livers in control group, **(B2)** IHC analysis of livers in inoculated group. The primary antibody was active caspase-9 antibody, and the positive signals were observed in the cytoplasm of hepatocyte (→); **(B3)** A semi-quantitative analysis of the ratio of active caspase-9 positive staining to the total field; **(B4)** Western-blot analysis of active caspase-9 protein in hepatocyte of control group and inoculated group gerbils; **(B5)** A semi-quantitative analysis of relative expression of active caspase-9/GAPDH. **(C1)** IHC analysis of livers in control group, **(C2)** IHC analysis of livers in inoculated group. The primary antibody was active caspase-3 antibody, and the positive signals were observed in the cytoplasm of hepatocyte (→); **(C3)** A semi-quantitative analysis of the ratio of active caspase-3 positive staining to the total field; **(C4)** Western-blot analysis of active caspase-3 protein in hepatocyte of control group and inoculated group gerbils; **(C5)** A semi-quantitative analysis of relative expression of active caspase-3/β-actin. ^∗^*p* < 0.05, ^∗∗^*p* < 0.01.

Immunohistochemical staining demonstrated that Apaf-1 expression in the hepatocyte cytoplasm of the inoculated group was significantly higher compared with that in the control group (**Figures 6A1,A2**). The up-regulation of Apaf-1 was confirmed by semi-quantitative analysis (^∗^*p* < 0.05 and ^∗∗^*p* < 0.01; **Figure 6A3**). Western-blot analyses showed that Apaf-1 expression was significantly higher in the inoculated group, compared with that in the control group (^∗^*p* < 0.05 and ^∗∗^*p* < 0.01; **Figures 6A4,A5**).

Active caspase-9 and caspase-3 were shown to be expressed at higher levels in the liver of HEV-infected gerbils, compared with those in the control group (**Figures 6B1,B2,C1,C2**). Similar findings were obtained in the semi-quantitative analyses (^∗^*p* < 0.05 and ^∗∗^*p* < 0.01; **Figures 6B3,C3**) and western-blot analyses (^∗^*p* < 0.05 and ^∗∗^*p* < 0.01; **Figures 6B4,B5,C4,C5**).

## Discussion

In this study, we determined that HEV infection of Mongolian gerbils may induce the ultrastructural and functional alterations in liver mitochondria, affecting their energy metabolism. ATPase and ATP synthetase, ATP5A1 levels were shown to decrease in the infected animals, together with SOD activity, while the concentration of MDA increased. Taken together, these data demonstrated that HEV infection induces mitochondrial injury, and, consequently, affects, the life cycle of hepatocyte (Everett et al., 2002; Canbay et al., 2004; Amacher, 2005; Guicciardi et al., 2013). TUNEL staining positive demonstrated an increase in apoptotic hepatocyte numbers in the HEV-infected animals, together with the increase in Bax, Bcl-2, Apaf-1, active caspase-3 and caspase-9 expression levels. Additionally, cytochrome c levels in the cytoplasm of the infected hepatocytes, in contrast to those in the hepatocytes obtained from the control group, showing that HEV infection induces hepatocyte apoptosis via mitochondrial pathway in Mongolian gerbils.

Gerbils were previously successfully infected with HEV, which was then detected in the infected livers, together with and the increased liver enzyme levels (Li et al., 2009; Yang et al., 2015). Here we analyzed the mechanisms underlying these effects and demonstrated that the hepatocyte mitochondria in the HEV-infected gerbils were considerably swollen, with thin cristae, indicating that mitochondria may be targeted during HEV infection.

Since mitochondria play a key role in the apoptotic cascades and cell death (Everett et al., 2002), we further analyzed the expression of mitochondrion-mediated apoptosis-associated proteins.

A previous study demonstrated that hepatocyte apoptosis may be induced by proinflammatory and profibrogenic stimuli, leading to the release of cellular constituents into serum, and liver injury (Canbay et al., 2004). Furthermore, hepatocytes are susceptible to the activation of the mitochondrial apoptotic pathway, in response to excessive free fatty acid generation in the metabolic syndrome (Guicciardi et al., 2013), while the HCV core protein was shown to induce hepatocyte injury and Huh7 cell mitochondrial pathways associated apoptosis (Chou et al., 2005). This pathway is regulated by the Bcl-2 family proteins (Cory and Adams, 2002; Youle and Strasser, 2008), which is why we examined the expression of the members of this family.

The swelling of mitochondrial and thinning of the cristae affects, synthesis and utilization of ATP. ATPase and ATP synthetase ATP5A1 levels in the infected hepatocytes decreased, indicating that HEV infection negatively infects ATP metabolism.

Superoxide dismutase is an important antioxidase, which plays a key role in maintaining the oxidative balance, and its activity levels indirectly reflect the ability of cells to remove free oxygen radicals (Knowles et al., 1969). Furthermore, MDA is a degradation product of lipid peroxidation, which occurs during the interaction between free radicals and poly unsaturated fatty acids (Janero, 1990), and MDA concentrations represent one of the biomarkers of the oxidative stress (Fogelman et al., 1980; Draper and Hadley, 1990). Oxidative stress contributes to the development of many diseases, including some age-related macular degenerations, which are attributed to an inadequately neutralization of oxidative stress (Del Priore et al., 2002; Dunaief et al., 2002; Schutt et al., 2003; Zhou et al., 2005; Weismann et al., 2011), and mitochondrial damage has been associated with oxidative stress related diseases (Chance et al., 1979; Grivennikova et al., 2010). A dysfunction of the mitochondrial antioxidant system may render mitochondria susceptible to injuries despite the adequate cytosolic antioxidant levels (Cano et al., 2014). Viral infections were shown to induce mitochondrial damage and oxidative stress (Acar et al., 2009; Khadem Ansari et al., 2015; Fan et al., 2016), which was confirmed in this study as well, since the SOD activity was shown to decreased while MDA concentrations increased in HEV-infected groups. Our combined results support the conclusion that HEV infection induces mitochondrial damage, which leads to oxidative stress development. Mitochondrial dysfunction may be directly or indirectly induced by HEV, and further studies are required to determine the specific mechanisms underlying this process.

To understand the pathogenesis of mitochondrial damage in hepatocytes induced by HEV infection, TUNEL staining was performed, and we determined the levels of proteins associated with the mitochondrial apoptosis pathway. We observed that the number of apoptotic hepatocytes increased in the HEV-infected animals. Furthermore, we analyzed the levels of Bax and Bcl-2, pro-apoptotic and anti-apoptotic proteins, respectively, since the Bax to Bcl-2 ratio plays an important role in the process of cell apoptosis (Adams, 2003). We demonstrated that the expression of Bcl-2 and Bax increased in the infected in the infected group. Bax acts downstream in the mitochondrial apoptotic pathway and it represents a key molecule in this process. As a component of the mitochondrial electron transfer chain, cytochrome c can initiate caspase activation following its release from mitochondria, binding to Apaf-1 (Du et al., 2015). Afterward, Apaf-1-cytochrome c complex forms apoptosome, which recruits procaspase-9 and initiates the formation of the caspase-9 holoenzyme that cleaves and activates downstream caspases, such as caspase-3. Furthermore, the recruitment of Bax leads to permeabilization of mitochondrial outer membrane (Wang, 2001; Du et al., 2015), which is considered one of the key control switches of the apoptotic process (Ferreira et al., 2008). Here, we demonstrated that cytochrome c was released into the cytosol in the inoculated group, and this represents a biomarker indicating the activation of the mitochondrial apoptotic pathway (Pan et al., 2014).

Currently, both animal models and cell cultures are available for HEV pathogenesis investigations. Our team established an efficient Mongolian gerbil model of HEV genotype 4 infection. HEVs were consistently detected in the liver, kidney, and other tissues of Mongolian gerbils, with the characteristic viral hepatitis lesions prominent in the liver. The concentration of AST, ALT, and T-BIL was shown to be significantly increased, while the ultrastructural hepatic injury and anti-HEV IgG positive seroconversion were observed during the infection (Li et al., 2009; Yang et al., 2015). HEV RNA was detected in the liver from 7 to 42 dpi, consistent with the last days of HEV infection in the swine (Bouwknegt et al., 2009), suggesting that the HEV RNA replication in the Mongolian gerbil was similar to its replication in the swine model. Previously, the activation of mitochondria and caspase-3 protease expression were shown to be induced, followed by the apoptosis and subsequent necrosis of renal epithelial cells, during the acute phase of HEV infection in Mongolian gerbils. An increased number of apoptotic cells were observed in the renal tubules of these animals, together with the increased levels of Bax, Bcl-2, and caspase-3 in the kidneys (Soomro et al., 2016). Furthermore, HEVs were detected in the testicular tissues, and the structural and molecular alterations leading to the disruption of the blood–testis barrier and germ cell apoptosis were observed (Soomro et al., 2017). Using this model, the HEV-4 was demonstrated to be able to cross the blood brain barrier and replicate in the brain and the spinal cord (Shi et al., 2016). Previously, a Z:ZCLA Mongolian gerbil model infected with HEVs isolated from an acute hepatitis E patient was established (Hong et al., 2015). These results are consistent with the observations of this study, and previous studies demonstrated that the Mongolian gerbil represent a promising model for the studies on HEV infection and pathogenesis (Doceul et al., 2016). *In vitro*, HEV cultivation was performed using PLC/PRF/5 and A549 using fecal suspension infected with HEV (Tanaka et al., 2007, 2009; Takahashi et al., 2010; Shen et al., 2014), and structural changes to the cells were investigated by TEM, showing that the lysosome numbers increased, indicating apoptosis, and cell membrane was damaged (Shen et al., 2014), which was also consistent with our research.

## Conclusion

Taken together, our results demonstrated that HEV infection induces the development of hepatocyte mitochondrion damage in the Mongolian gerbils. Mitochondria were in the oxidative stress situation and were shown to be considerably swollen and their energy metabolism was disrupted. Our investigations demonstrated that HEV infection activated the mitochondrial apoptotic pathway, which triggered the hepatocyte apoptosis.

## Author Contributions

YY and RPS were responsible for the study design. YY, RHS, MS, FH, and FD performed the laboratory work. YY analyzed the data and wrote the article. All authors read, commented on and approved the final article.

## Conflict of Interest Statement

The authors declare that the research was conducted in the absence of any commercial or financial relationships that could be construed as a potential conflict of interest.

## Abbreviations

ALT: alanine transaminase
AST: aspartate transaminase
ATP: adenosine triphosphate
Bax: Bcl2-associated X protein
dpi: day post-inoculation
HEV: hepatitis E virus
MDA: malondialdehyde
ORFs: open reading frames
SOD: superoxide dismutase
SPF: specific-pathogen free
TBIL: total bilirubin
TEM: transmission electron microscopy
TUNEL: TdTmediated dUTP nickend labeling
